# Simple, green, ultrasound-assisted preparation of novel core–shell microcapsules from octyl methoxycinnamate and oligomeric proanthocyanidins for UV-stable sunscreen†

**DOI:** 10.1039/d0ra09116b

**Published:** 2021-02-04

**Authors:** Jie Song, Siqi Chen, Xu Zhao, Junbo Cheng, Yanli Ma, Shixue Ren, Shujun Li

**Affiliations:** Key Laboratory of Bio-Based Material Science & Technology (Northeast Forestry University), Ministry of Education Harbin 150040 China renshixue@nefu.edu.cn

## Abstract

Without sunscreens, UV rays in sunlight cause skin damage, ranging from dark spots and premature aging to skin cancer. Present sunscreens, however, are readily photodegraded, producing highly reactive radicals that can damage cells. To address this problem, we have now used ultrasound to prepare core–shell microcapsules, which offer improved protection against UV light and improved UV stability. The composite microcapsules have oligomeric proanthocyanidins (OPCs), which are amphiphilic plant-derived secondary metabolites, as the shell and octyl methoxycinnamate (OMC), which is a UVB absorber, as the core. The polyphenolic flavonoid structure of OPCs improves the UV stability of OMC and thus avoids the skin damage caused by OMC photodegradation products. In the microcapsules, π–π stacking interactions between OPCs and OMC molecules enhance the ability of OMC to absorb UV radiation and extend the absorption range from the UVB region (280–320 nm) to include the UVA and UVC regions (200–400 nm). The composite microcapsules were shown to be stable on storage and to be non-irritant to human skin. The ultrasound-assisted preparation of OMC/OPCs composite microcapsules is simple, efficient and green and provides a feasible strategy for the development of novel, more effective, sunscreens.

## Abstract 2

With oligomeric proanthocyanidins (OPCs) as the outer shell and ultraviolet absorbers (OMC) as the inner core, OMC/OPCs composite microcapsules were prepared and characterized, and their UV resistance was studied.

## Graphical abstract

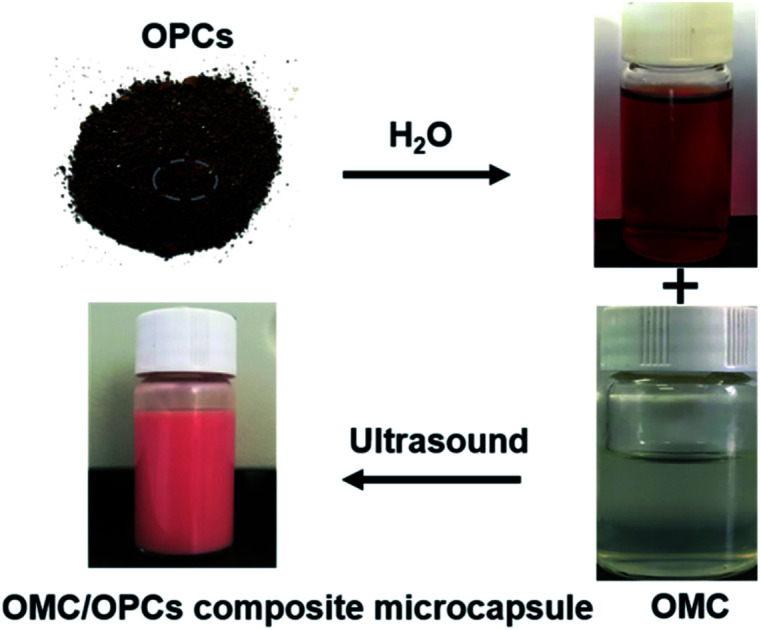

## Introduction

With recent improvements in living standards and more time for outdoor leisure activities, there is increased demand for effective sunscreens to prevent skin damage.^1,2^ Sunscreens can be divided into physical sunscreens, which typically block ultraviolet (UV) light by reflection or scattering,^3–5^ and chemical sunscreens, which typically contain molecules that absorb harmful UV irradiation.^6,7^ Agents commonly used in physical sunscreens include zinc oxide and titanium dioxide, and such preparations leave the skin feeling unpleasantly greasy after application.^8–11^ Chemical sunscreens, on the other hand, usually produce a pleasant feeling, but the molecules that absorb UV radiation can produce free radicals,^12^ which lead to accelerated aging, allergies and even skin cancer.^10–13^ Improving the stability of chemical sunscreen agents to UV radiation is thus an important goal.^14–17^

Octyl methoxycinnamate (OMC), which is widely used in chemical sunscreens, is lipid soluble and has a good safety profile.^18^ OMC is a *para* methoxy-substituted cinnamic acid derivative. Its extended conjugated π bonded structure allows OMC to absorb UV light,^19^ especially in the region 280–310 nm, but OMC is also readily degraded by UV light,^20^ producing highly reactive radicals that can destroy biological macromolecules and damage cells.^20–24^ Another downside of OMC is its poor stability in water/alcohol solutions, to some extent, which limits its use in the cosmetics and sunscreen industries.^25–27^

Two strategies can be considered for improving the stability and performance of OMC. The first strategy is to use additives to inhibit UV degradation of OMC. Flavonoids have been reported to effectively inhibit UV degradation of molecules used in chemical sunscreens.^28–30^ Larch bark extract contains a large number of polyphenols (*e.g.*, flavonoids). The main components of flavonoids are tannins and phenolic acids shaped by proanthocyanidins. Procyanidins are formed by combining different amounts of catechins or epicatechins, it can be divided into higher molecular weight polymers (*n* ≥ 5, polymeric proanthocyanidins, PPCs) and lower molecular weight polymers (*n* < 5, oligomeric proanthocyanidins, OPCs). Oligomeric proanthocyanidins are usually formed by oligomers composed of catechin, epicatechin, or both. (Such as dimer standard proanthocyanidins B1). OPCs, which have less steric hindrance than PPCs, are better able to inhibit UV photodegradation of sunscreen molecules and also have excellent antioxidant and biological activities.^31^

The second strategy is to avoid direct skin contact with the sunscreen and UV degradation products, which can be achieved by preparing the sunscreen in the form of microcapsules.^32–34^ The methods for preparing microcapsules can be broadly divided into physical methods, chemical methods and physical–chemical methods. Physical methods are inexpensive and are widely used in current manufacturing processes. Ultrasonic fields are often used to facilitate the preparation of microcapsules by physical methods. It is generally believed that cavitation effects,^35^ mechanical effects, thermal effects and chemical effects all take place when ultrasound propagates in the medium.^36–38^ In ultrasound-assisted preparation of microcapsules, ultrasound explosively destroys the tiny bubbles formed by acoustic vibration in the medium. When these bubbles collapse, they release huge energy, forming local shear forces and leading to dispersion and emulsification of the system. The polymeric molecules that make up the microcapsule wall form a network structure at the air or phase boundary and encapsulate the core material, in a process that is also known as the ultrasonic field-induced cavitation effect.^39,40^ For example, Li and others have used ultrasound cavitation to synthesize multifunctional bovine serum albumin microcapsules for targeted drug delivery and controlled release. Zhou *et al.* used the same method to prepare lysozyme microspheres encapsulating different types of liquids and found that the type of encapsulated liquid affected the physical and chemical properties of the microspheres. Zhang *et al.* prepared magnetic dual-targeted folate–cysteine–Fe_3_O_4_ microcapsules with redox properties using ultrasound. Folic acid and Fe_3_O_4_ magnetic nanoparticles were successfully introduced into the microcapsule shell.^41–43^

Ultrasound-assisted preparation of microcapsules is simple, efficient and green. Here, we used ultrasound to facilitate the preparation of octyl methoxycinnamate/oligomeric proanthocyanidins (OMC/OPCs) composite microcapsules. We explored the effects of added surfactant, ultrasound-assisted reaction time, ultrasonic power, OPCs mass concentration and OMC to OPCs mass ratio on the particle size distribution and average particle size of the microcapsules. The microcapsules were characterized using Fourier-transform infrared (FTIR) spectroscopy and scanning electron microscopy (SEM). The resistance to UV degradation, storage stability and safety of the OMC/OPCS microcapsules on human skin were also investigated. Larch bark contains as much as 10–16% proanthocyanidins by weight, and the amount of larch bark waste produced in China approaches 90 000 tons per year. This study not only provides a way to enhance the value of OPCs obtained from larch bark, but also expands their application into the fields of sunscreens and skin care.

## Results and discussion

### The composition of the oligomeric proanthocyanidins (OPCs) isolated was analyzed

The OPCs and proanthocyanidin B1 were characterized by hydrogen nuclear magnetic resonance spectroscopy (NMR), as shown in Fig. S1.† (The standard proanthocyanidins is a typical dimer composed of catechin and epicatechin.) The ^1^H NMR spectra of OPCs and proanthocyanidins B1 show peaks at 1.9 ppm, 2.5–3.3 ppm, 3.6–4.4 ppm, 4.6–5.0 ppm, 5.7–6.0 ppm, 6.7 ppm, 7.7–8 ppm and 8.8–9.2 ppm.^53^ The spectrum of OPC is similar to those of proanthocyanidins B1, showing that the structure of the extracted product are similar to those of proanthocyanidin B1.

### Proposed mechanism preparation of OMC/OPCs composite microcapsules

**Fig. 1:**
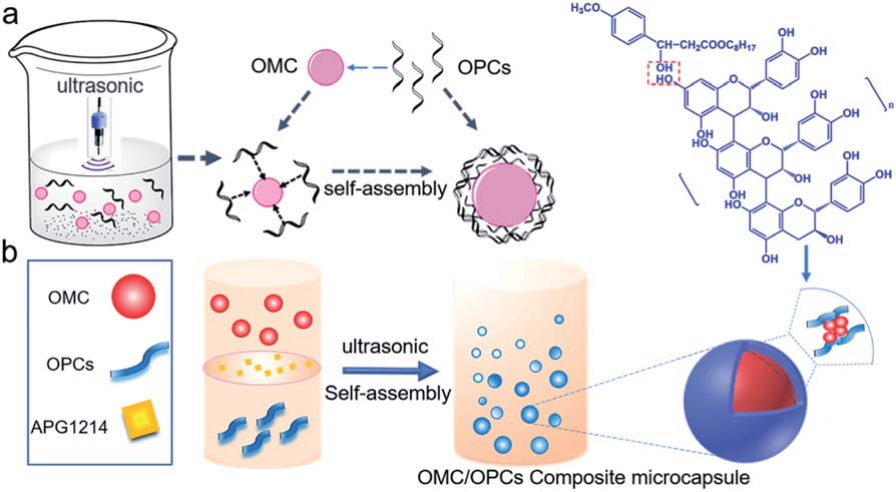
(a and b) Mechanism of preparation of OMC/OPCs composite microcapsules.

Ultrasound facilitates the emulsification and dispersion of OMC and OPCs. In the presence of surfactants, OPCs molecules coat the OMC layer by layer. Macroscopically, homogeneous OMC/OPCs microemulsions was formed. OPCs are amphiphilic low molecular weight polymers that contain hydrophobic benzene rings and hydrophilic phenolic hydroxyl groups and aliphatic hydroxyl groups. Under the influence of ultrasound, OPCs self-assemble layer by layer to form a network structure that encapsulates the OMC, as shown in Fig. 1a.

The ultrasonic field also causes cavitation, which forces active sites in the OPCs molecules, such as the phenolic hydroxyl groups, to combine more closely with the OMC through intermolecular hydrogen bonds. The resulting OMC/OPCs composite microcapsules have an obvious shell structure, which prevents leakage of OMC and its photodegradation products from the microcapsules,^44–46^ as shown in Fig. 1b. The FTIR spectrum of the OMC/OPCs microcapsules was largely similar to that of OPCs (Fig. 4f), indicating that no new chemical bonds had been formed in the process.

#### Influence of surfactants Tween 80 and APG1214

The effects of using a surfactant (Tween 80 or APG1214), compared with no surfactant, on the particle size of the composite microcapsules were explored using the following reaction conditions: ultrasound-assisted reaction time 5 min; ultrasonic power of 400 W; mass concentration of OPCs of 2%; OMC to OPCs mass ratio of 1 : 1.

**Fig. 2:**
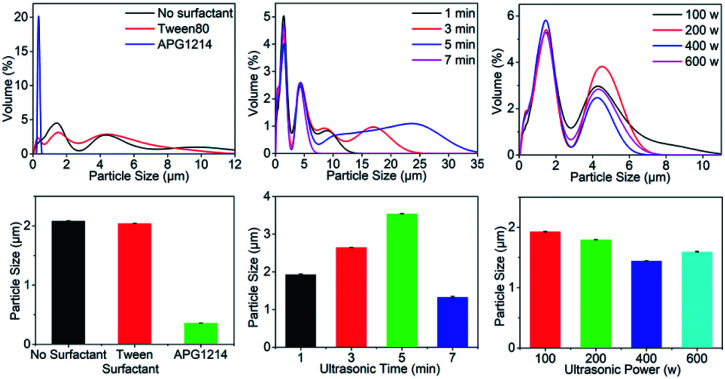
(a, c and e) Effects of surfactant, ultrasonic time and ultrasonic power on particle size distribution of OMC/OPCs composite microcapsules. (b, d and f) Effects of surfactant, ultrasonic time and ultrasonic power on average particle size of OMC/OPCs composite microcapsules.

The particle size distribution of composite microcapsules prepared in the presence of Tween 80 or APG1214, or with no surfactant, is shown in Fig. 2a. The size distribution of the microcapsules was greatest when no surfactant was added and, after ultrasonic treatment, the solution was uniform. In the presence of Tween 80, the distribution range of microcapsule sizes in the emulsion was reduced to less than 12 μm and, in the presence of the same quantity of APG1214, the distribution range of particle sizes became much narrower and was concentrated around 0.5 μm. The average particle size of the composite microcapsules prepared under different surfactant conditions confirmed this result (Fig. 2b). In the presence of APG1214, the average particle size of the microcapsules was smaller than when Tween 80 or no surfactant was added.

Generally speaking, surfactants reduce the interfacial tension between water and oil, and contribute to the formation and stability of microemulsions. From a structural point of view, both APG1214 and Tween 80 are non-ionic surfactants. In the OMC/OPCs system, the hydrophilic end of the surfactant is compatible with OPCs molecules, and the hydrophobic end is bound to the oily OMC molecules. The emulsification of the system is greatly enhanced by reducing the surface free energy and surface tension at the interface. The average particle size of the OMC/OPCs composite microcapsules was in the order of microns. APG1214 has the characteristics of ordinary nonionic and anionic surfactants, has higher surface activity, is non-toxic, harmless, non-irritating to the skin, and biodegrades rapidly. It is thus more suitable for increasing the dispersion and stability of OMC/OPCs composite microcapsule and was used as the surfactant in subsequent experiments.

#### Effect of ultrasound-assisted reaction time

In addition to the effects of surfactants, the ultrasonic reaction time, power, OPCs concentration, OMC to OPCs mass ratio and other factors will also affect the particle size of the composite microcapsules. The effects of ultrasound-assisted reaction time on the particle size of composite microcapsule was investigated using the following reaction conditions: ultrasonic power (200 W); OPCs mass concentration (2%); OMC to OPCs mass ratio (1 : 1); APG1214 to OMC mass ratio of 1 : 1.

There was no significant difference in the particle size distribution of the composite microcapsules when the ultrasound-assisted reaction time was in the range 1–7 min (Fig. 2c). The average particle size of the composite microcapsules first increased, and then decreased, as the reaction time increased (Fig. 2d). The maximum particle size of 3.537 μm was achieved with a reaction time of 5 min. When the reaction time was extended to 7 min, the average particle size showed a sudden decrease, and was smaller than the average particle size formed with a reaction time of 1 min. This is because, with increased reaction time (1 min–3 min–5 min) the thermal effect of the ultrasonic field increases the probability of interparticle collisions, leading to an increase in the average particle size of the microcapsules. When the reaction time is very short (1 min), the formation of OMC/OPCs composite microcapsules is not sufficient, leading to failure of the OPCs to fully emulsify OMC. When the reaction time was 5 min, the particle size distribution curve showed characteristics of small particle concentration and large particle dispersion, and the average particle size was the largest. This may be caused by austenitic maturation (instability in which small particles disappear and large particles grow). When the reaction time was 7 min, the average particle size of the microcapsules suddenly decreased and became smaller than the average particle size formed with a reaction time of 1 min. This is because continued exposure to ultrasound breaks up the composite microcapsules that have already formed. The best ultrasonic reaction time is thus 3 min.

#### Influence of ultrasonic power

The effect of ultrasonic power on the particle size of OMC/OPCs composite microcapsules was investigated using the following reaction conditions: ultrasound-assisted reaction time 3 min; OPCs concentration of 2%; OMC to OPCs mass ratio of 1 : 1; OMC to APG1214 mass ratio of 1 : 1.

The particle size distribution curves of the composite microcapsules were similar when different ultrasonic powers were used. The particle sizes were concentrated around 1 μm and 4.2 μm, and the amount of 1 μm particles was largest at 400 W (Fig. 2e). As the ultrasonic power increased, the average particle size of the composite microcapsules first decreased and then increased (Fig. 2f). Generally speaking, with the same ultrasound-assisted reaction time, the higher the ultrasonic power, the better the ultrasonic dispersion of the OMC/OPCs system, which is manifest as a smaller average particle size. With increasing ultrasonic power, the cavitation effect caused by ultrasonic resonance in the emulsion system is increased, promoting dispersion of the proanthocyanins at the water/oil interface. This reduces the particle size of the composite microcapsules and makes the entire OMC/OPCs system more uniform and stable. When the ultrasonic power is too high, the cavitation effect forces the OMC/OPCs system into a high temperature state and destroys the molecular structure of individual components, or the structure of the composite microcapsule wall, and reduces the performance of the system. The most appropriate ultrasonic power is thus 400 W.

#### Influence of OPCs mass concentration

**Fig. 3:**
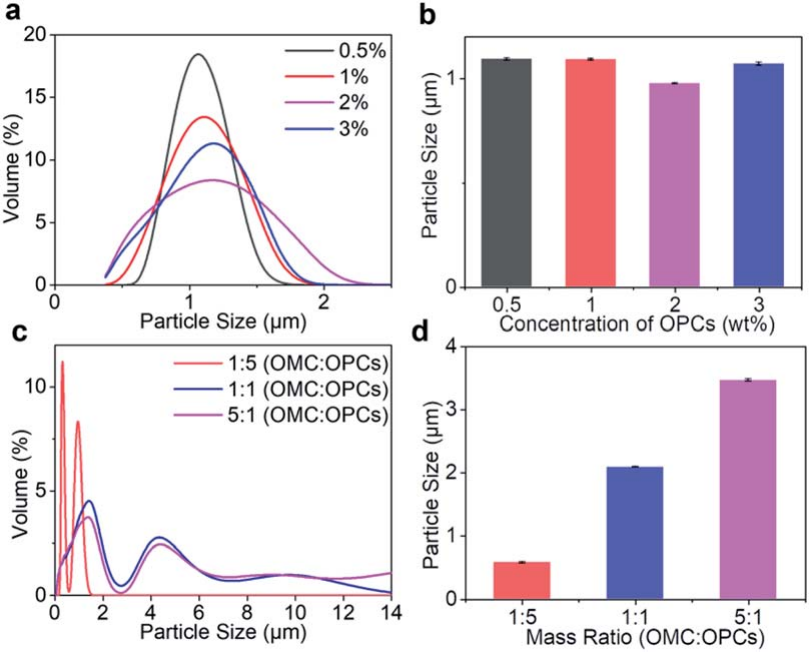
(a and c) Effects of OPCs concentration, OMC to OPCs mass ratio on particle size distribution of OMC/OPCs composite microcapsules. (b and d) Effects of OPCs concentration and mass ratio of OMC to OPCs on average particle size of OMC/OPCs composite microcapsules.

The influence of OPCs mass concentration on microcapsule particle size was investigated using the following conditions: ultrasound-assisted reaction time 3 min; ultrasonic power of 400 W; OMC to OPCs mass ratio of 1 : 1; OMC to APG1214 mass ratio of 1 : 1. At mass concentrations of 0.5%, 1%, 2% and 3%, combined with Fig. 3a and b shows that the mass concentration of OPCs had little effect on the approximate particle size distribution and average particle size of the composite microcapsules.

#### Influence of OMC to OPCs mass ratio

The influence of OMC to OPCs mass ratio on the particle size of the composite microcapsule was investigated using the following conditions: ultrasound-assisted reaction time 3 min; ultrasonic power of 400 W; OPCs mass concentration of 2%; OMC to APG1214 mass ratio of 1 : 1. With increasing OMC to OPCs mass ratio, the particle size distribution curve of the composite microcapsules becomes wider (Fig. 3c). When the OMC to OPCs mass ratio was 1 : 5, the particle size was in the range 0–2 μm, and was mainly concentrated at about 0.5 μm. When the mass ratio was 1 : 1 or 5 : 1, composite microcapsules with larger particle sizes appeared, and the distribution of particle sizes was wider at a mass ratio of 5 : 1. With increasing OMC to OPCs mass ratio, the average particle size of OMC/OPCs composite microcapsules also increased significantly (Fig. 3d).

### Morphology of OMC/OPCs composite microcapsules

**Fig. 4:**
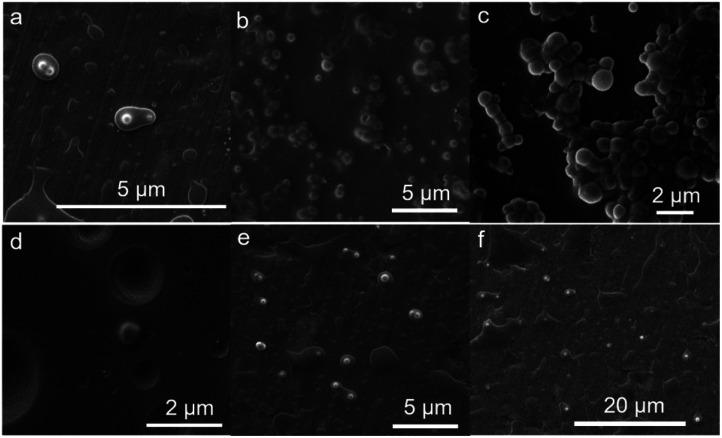
(a–c) SEM image of OMC/OPCs composite microcapsule with different OMC to OPCs mass ratios of 1 : 5, 1 : 1 and 5 : 1. (d) SEM images of OMC/OPCs composite microcapsules prepared under optimum conditions. (e) Scanning electron microscope image without surfactant. (f) Microcapsule morphology under very diluted conditions.

In order to explore the reasons for these observations, we used SEM to observe the microscopic morphology of the composite microcapsules prepared using different OMC to OPCs mass ratios. In general, the greater the mass ratio of OMC to OPCs, the more marked the aggregation of the composite microcapsules. When the OMC to OPCs mass ratio was 1 : 5, *i.e.*, when more OPCs was present, the OMC molecules in the emulsion were coated with OPCs and took on a polynuclear shape (Fig. 4a). When the mass ratio was 1 : 1, the OMC/OPCs composite microcapsule had a spherical structure, with smooth surfaces and a relatively uniform dispersion (Fig. 4b). When the mass ratio was 5 : 1, excess OMC was present, which destroyed the water–oil balance and caused the microcapsules to accumulate and form clusters (Fig. 4c), giving the appearance of a larger particle size. An OMC/OPCs mass ratio of 1 : 1 is thus most suitable for the preparation of composite microcapsules.

In the preceding section, we have discussed the effects of surfactants, ultrasound-assisted reaction time, ultrasonic power, OPCs concentration, OMC to OPCs mass ratio and other factors on the particle size of OMC/OPCs composite microcapsules. We determined that the best conditions for the preparation of OMC/OPCs composite microcapsules were: ultrasound-assisted reaction time 3 min; ultrasonic power of 400 W; OPCs to OMC mass ratio of 1 : 1, OPCs mass concentration of 2%; OMC to APG1214 mass ratio of 1 : 1. OMC/OPCs composite microcapsules prepared using these conditions had the best particle size and were used in the following studies. SEM images of OMC/OPCs composite microcapsules, prepared under optimum conditions, are shown in Fig. 4d. The microcapsules are uniformly distributed and have a smooth surface, and the core and wall material layers of the composite microcapsules can be seen to have an obvious core–shell structure. (The shell thickness of the microcapsules is roughly calculated to be between 0.4–0.5 μm.) In addition, we performed scanning electron microscopy tests on the microcapsules under extremely diluted conditions (Fig. 4f) and found that the morphology of the microcapsules can also be seen in this case. The formation of microcapsules is also affected by surfactants. In order to rule out whether the surfactant is a single shell layer of OMC, the samples without surfactant APG1214 were tested by SEM and TEM (Fig. 4e and S3†). It was found that the morphology of microcapsules can still be observed without surfactant addition.

### FTIR and XPS analysis

**Fig. 5:**
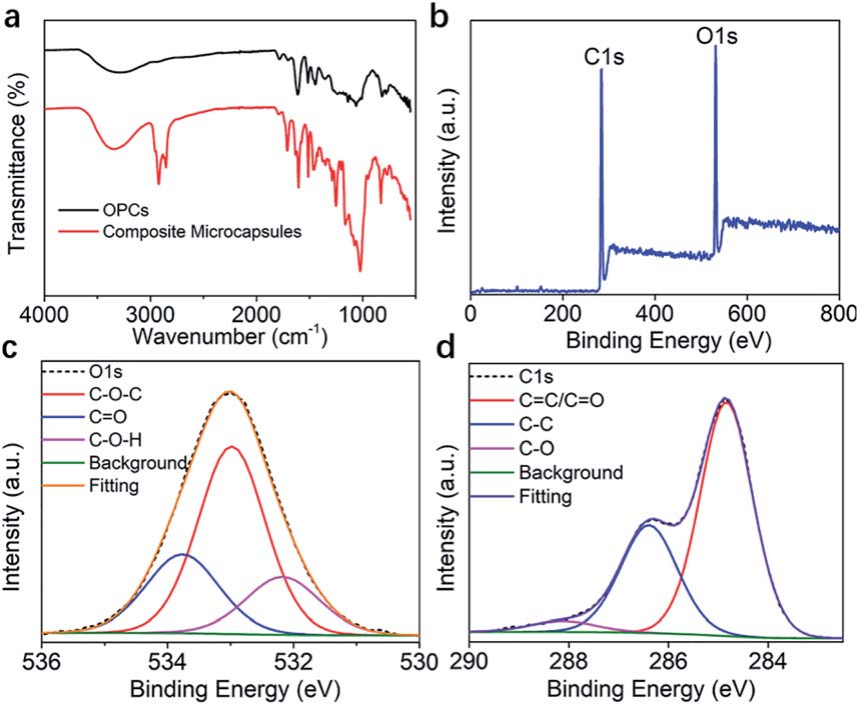
(a) FTIR spectra of OPCs and OMC/OPCs composite microcapsules; (b) full XPS spectrum of OMC/OPCs composite microcapsules; (c and d) O 1s and C 1s XPS spectra of OMC/OPCs composite microcapsules.

The FTIR spectra of OPCs and OMC/OPCs composite microcapsules are shown in Fig. 5a. Both spectra contain characteristic absorption peaks, including the stretching vibration of phenolic O–H at 3300 cm^−1^, the respiratory vibrations of benzene ring skeleton C=C double bond at 1600 cm^−1^, 1580 cm^−1^, 1500 cm^−1^ and 1450 cm^−1^, the stretching vibration of C–O–C at 1300 cm^−1^, and the out-of-plane bending vibration of unsaturated C–H at 800 cm^−1^.

The peak intensities of the OMC/OPCs composite microcapsules were enhanced to different degrees compared with those of OPCs, because of increased aromatic functional groups and ether bonds after addition of OMC. Compared with OPCs, the OMC/OPCs composite microcapsules showed sharper characteristic absorption peaks at 2885 cm^−1^, 2770 cm^−1^ and 1750 cm^−1^ because of the presence of –CH_3_, –CH_2_–, –CH, and C=O functional groups in the OMC molecules.

XPS was used to further investigate the chemical composition of the OMC/OPCs composite microcapsules and OPCs. The full XPS survey spectrum both of them indicated the presence of C and O (Fig. 5b and S4a†).^51,52^ In the high-resolution O 1s spectrum, there are 3 and 2 peaks in microcapsules and OPCs, respectively (Fig. 5c and S4b†). O1s spectrum of microcapsules (Fig. 5c) showed peaks at 532.9, 533.7, 532.2 eV, which was attributed to C–O–C/C=O/C–O–H (the C=O bond comes from the OMC in the microcapsule). and the peaks of OPCs at 532.1, 533 eV can be observed in Fig. S4b,† they are C–O–H/C–O–C. The C 1s spectrum (Fig. 5d) showed peaks at 284.8, 286.73 and 288.1 eV (OMC/OPCs composite microcapsules), which were assigned to C=C(C=O)/C–C/C–O, respectively. OPCs has peaks at 283.8 eV (C=C), 285.2 eV (C–C), 288.1 eV (C–O) seen from the C 1s spectrum (Fig. S4c†). The XPS spectra thus indicated that no new chemical bonds are formed, consistent with the FT-IR.

### UV resistance of OMC/OPCs composite microcapsules

**Fig. 6:**
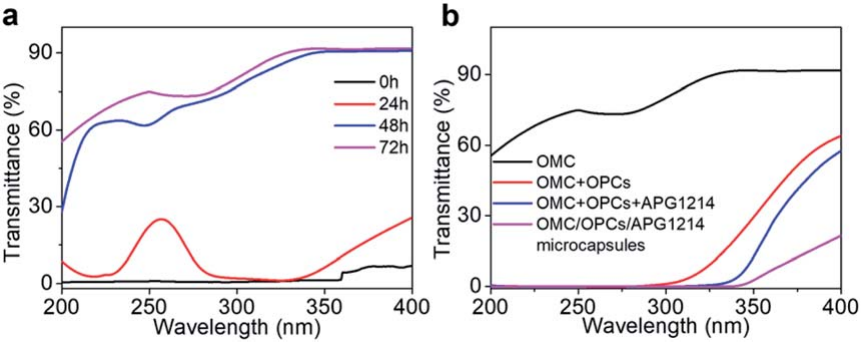
(a) Effect of xenon lamp exposure time on OMC degradation. (b) UV transmittance of OMC, OMC + OPCs blend, OMC + OPCs + APG1214 blend (no ultrasound treatment) and OMC/OPCs/APG1214 composite microcapsules after irradiation for 72 h.

OMC is one of the mostly commonly used UV filters in sunscreens. Although OMC has a good shielding effect against UVB radiation (280–320 nm), it has only a weak shielding effect against UVC (200–280 nm) and UVA (320–400 nm) radiation. OMC is also prone to degradation under the action of UV light. A solution of OMC was irradiated with a xenon lamp for 24 h, 48 h and 72 h under the same conditions and the UV transmittance of the solution was recorded using a UV spectrophotometer to observe the degradation of OMC (Fig. 6a). The UV transmittance of un-irradiated OMC (0 h) was used as a control.

After 24 h of continuous irradiation with the xenon lamp, the UV transmittance of the OMC solution in the UVB region had increased by only 1–2%, indicating that most of the OMC molecules were not degraded at this time and could still effectively absorb UV light in the UVB region. After irradiation for 48 h and 72 h, the OMC solution had higher transmittance over the entire ultraviolet region (200–400 nm). UVB region. After irradiation for 48 h and 72 h, the OMC solution had higher transmittance over the entire ultraviolet region (200–400 nm). With increased irradiation time, the molecular structure of OMC undergoes significant photodegradation and the UV shielding effect is much reduced.

We next investigated whether addition of OPCs and APG1214 could inhibit the photodegradation of OMC. The OMC + OPCs and OMC + OPCs + APG1214 blends used here were prepared by direct blending, with no ultrasound treatment. The UV transmittance of OMC, OMC + OPCs blend, OMC + OPCs + APG1214 blend and OMC/OPCs/APG1214 composite microcapsules was measured after 72 h irradiation with the xenon lamp (Fig. 6b).

**Fig. 7:**
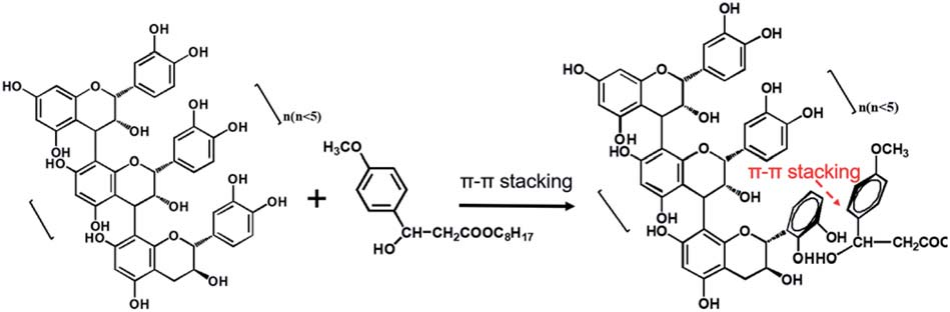
Schematic diagram of π–π* stacking of OPCs and OMC molecules.

After 72 h irradiation with the xenon lamp, OMC was significantly degraded and did not provide good UV-blocking over the entire UV region (200–400 nm). The OMC + OPCs blend had a good shielding effect against both UVC (200–280 nm) and UVB (280–320 nm) radiation. Addition of OPCs not only greatly improved the shielding effect over the entire UV region, but also inhibited the photodegradation of OMC. There are two main reasons for this. Firstly, OPCs are flavonoids with a large number of phenolic hydroxyl groups, which results in strong UV absorption around 280 nm. Secondly, both OPCs and OMC contain a large number of conjugated π bonds, which easily generate π–π* stacking (π–π* stacking of OPCs and OMC as shown in Fig. 7).^47,48^ This stacking effect will further enhance absorption of UV light in the region 280–310 nm, which is manifest as a decrease in UV light transmission. (That is, there is synergy between them).^49,50^

Under the same conditions, the OMC + OPCs + APG1214 blend absorbs UV light more effectively than the OMC + OPCs blend. This may be because introduction of the surfactant APG1214 results in more uniform mixing of the water and oil phases, leading to a decrease in interfacial tension and gradual disappearance of the phase interface.

The OMC/OPCs/APG1214 composite microcapsules prepared by the ultrasound method clearly had the best ability to absorb UV light in the region 200–400 nm (Fig. 6b). This is because, in addition to the π–π* stacking between OMC and OPCs molecules, the core–shell structure of the wall material of the microcapsules can also protect OMC molecules in the core from damage. The OMC/OPCs/APG1214 composite microcapsules thus inhibit degradation of the chemical sunscreen OMC, while showing excellent UV absorption.

In summary, the OMC/OPCs composite microcapsules prepared using ultrasound have two advantages. Firstly, the microcapsules provide optimal UV absorption and inhibition of OMC degradation. Secondly, the UV protection of the OMC/OPCs composite microcapsules has expanded from UVB (280–320 nm) to UVA (320–400 nm).

### Stability of OMC/OPCs composite microcapsules on storage

**Fig. 8:**
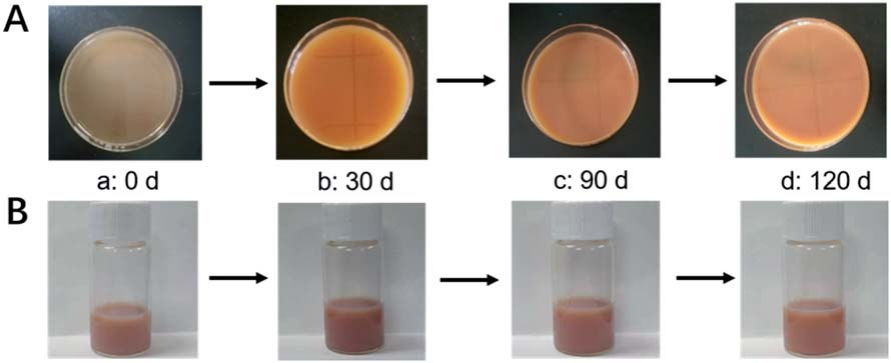
Effect of time on the storage stability of OMC/OPCs composite microcapsules. (A: front view; B: side view).

According to Stokes's law, the particle size of an emulsion in the gravity field is closely related to the settling velocity, *i.e.*, the smaller the particle size, the lower the probability of precipitation. However, if the particle size is too small, particles in the emulsion tend to agglomerate and accumulate over time, resulting in emulsion breakage and precipitation. Here, the storage stability of the OMC/OPCs composite microcapsules was tested by storing the microcapsules in glass Petri dishes at room temperature and the camera was used to take the front view and side view every 30 days, and the morphological changes were recorded (Fig. 8A and B). After 4 months of storage, the OMC/OPCs composite microcapsules did not show any macroscopic demulsification or precipitation, indicating that the samples have good storage stability.

### Safety of OMC/OPCs composite microcapsules

**Fig. 9:**
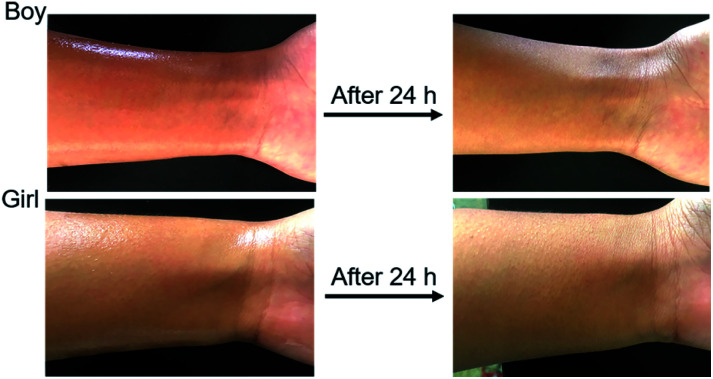
Surface condition of human skin (boy and girl) 24 h after application of microcapsules.

To test the safety of OMC/OPCs composite microcapsules on human skin, a microcapsule emulsion, prepared under optimal conditions, was evenly applied to the skin on the inner side of the left wrist of the test subject and the surface condition of the skin was observed after 24 h. There was no visible change in the skin and no allergic reaction (Fig. 9), showing that there is little or no irritation of human skin by the OMC/OPCs composite microcapsules. Although further verification is needed, this preliminary experiment shows that OMC/OPCs composite microcapsules are safe for use on human skin.

## Conclusion

OPCs have a polyphenolic structure and amphiphilic properties and can be used to coat the lipid-soluble UV absorber, OMC. OMC/OPCs composite microcapsules, which have a core–shell structure, were prepared using ultrasound, and the preparation process was optimized. Adding OPCs to form OMC/OPCs composite microcapsules was shown to improve the UV stability of OMC and to avoid skin damage caused by OMC photodegradation products. OPCs form π–π* stacking structures with OMC molecules, which further enhances the ability of OMC to absorb UV light and both improves the UV shielding effect and expands the scope of UV protection. OMC/OPCs composite microcapsules also have good storage stability and are safe to use on human skin.

The ultrasound-assisted preparation of OMC/OPCs composite microcapsules is simple, efficient and green and expands the application of larch bark OPCs into the field of sunscreen and skin care. This value-added application of larch bark OPCs merits further attention.

## Experimental

### Materials

Crushed larch bark was obtained from Inner Mongolia and crushed to a particle size of 0.5–1.0 mm. Analytical grade ethanol, petroleum ether and ethyl acetate were purchased from Tianjin Tianli Chemical Reagent Co., Ltd. (Tianjin, China). Octyl methoxycinnamate (octinoxate, OMC) was provided by Qingdao Yousuo Chemical Technology Co., Ltd. (Qingdao, China) and its purity is 95%. Alkyl polyglucoside (APG1214) and Polysorbate 80 (Tween80) were provided by Shandong Yousuo Chemical Technology Co., Ltd. (Shandong, China). The content of them are 50% and 99% respectively. Ultrapure water was generated using a Clever-Q30 UT ultrapure water system (Zhiang Instrument Co., Ltd., Shanghai, China).

### Extraction of OPCs from larch bark

Pulverized larch bark (150 g) was refluxed with aqueous ethanol (70% (v/v), 1500 mL) for 2 h and the mixture was then filtered under suction. The solid residue was refluxed with aqueous ethanol (70% (v/v), 1000 mL) for 1 h and the mixture was filtered. The combined filtrates were washed with an equal volume of petroleum ether and then allowed to stand for 15 min to allow impurities, such as gum and resin, to settle. The purified ethanol solution was decanted and the ethanol was removed by rotary evaporation (bath temperature 45 ± 5 °C). The resulting aqueous solution was extracted six times with an equal volume of ethyl acetate. The ethyl acetate extracts, which contained the OPCs, were combined and evaporated using a rotary evaporator (bath temperature 55 ± 5 °C). The residue was vacuum dried at (45 ± 5 °C) to provide larch bark OPCs as a powder.^53,54^

### Preparation of OMC/OPCs composite microcapsules

**Scheme 1:**
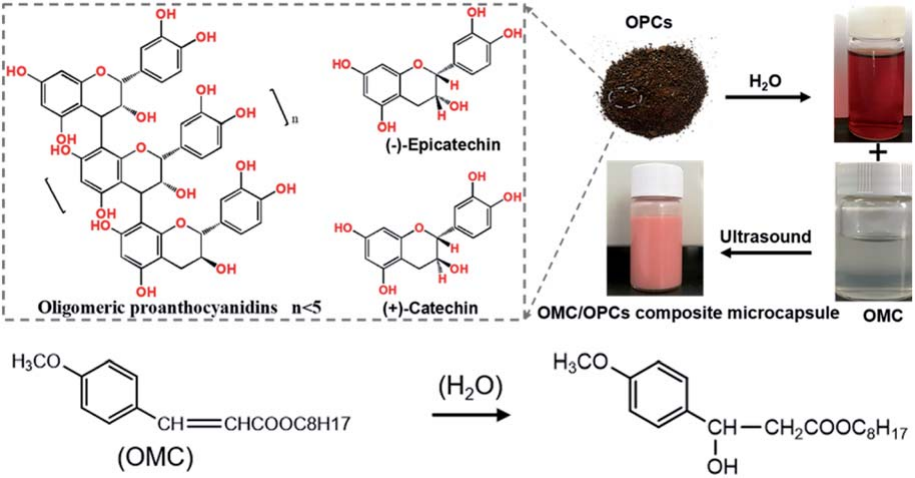
Preparation of OMC/OPCs composite microcapsules and the structure of OMC.

OMC/OPCs composite microcapsules were prepared using ultrasound. OPCs (0.2 g) were dissolved in ultrapure water (10 mL) by stirring at room temperature for 24 h. The solution was filtered through a 0.45 μm aqueous phase filter membrane and treated with different amounts of OMC and surfactants (APG1214 or Tween 80), as described below, to give a mixed emulsion. After stirring at room temperature for 30 min, the emulsion was subjected to ultrasound using a JY92-IIN ultrasonic cell pulverizer (Ningbo Xinzhi Biotechnology Co., Ltd.). Different ultrasonic power and reaction times were investigated, as described below, to determine the best conditions for forming an emulsion of OMC/OPCs composite microcapsule. The preparation process is shown in Scheme 1.

### Characterization of OMC/OPCs composite microcapsules

#### Analysis of particle size

The OMC/OPCs composite microcapsule emulsion was washed with ultra-pure water and centrifuged at 8000 rpm for 15 min. The microcapsules were then dispersed using a KQ-200VDE ultrasonic cleaning machine (Kunshan Ultrasonic Instrument Co., Ltd., Ningbo, China) for 1 min, at a power of 100 W. This process was carried out three times or more times, until the particle size distribution of the microcapsules tends to be consistent. (Fig. S2†) The particle size distribution of the OMC/OPCs composite microcapsules was then determined using an LS13320 laser particle size analyzer (Beckman Coulter, California, USA). Deionized water was used as the dispersant and the particle size distribution was recorded when the laser particle size analyzer displayed a concentration of 30%.

#### Scanning electron microscopy

A small amount of OMC/OPCs composite microcapsule emulsion was diluted with ultrapure water and dropped onto aluminum foil. The sample was allowed to air dry naturally at room temperature and then sprayed with gold. The morphology of the microcapsules was observed using a Quanta 200 scanning electron microscope (FEI Company, Eindhoven, Netherlands), with a field emission voltage of 12.5 kV and magnification of 5000.

#### Fourier-transform infrared spectroscopy

A small amount of OMC/OPCs composite microcapsule emulsion was freeze-dried using a Scientz-10ND freeze-dryer (Ningbo Xinzhi Technology Co., Ltd., Ningbo, China). The FT-IR spectrum of the freeze-dried sample was recorded over the range 4000–400 cm^−1^, using an FT-IR spectrometer (Perkin Elmer co., Waltham, USA). The resolution was 4 cm^−1^ and the sample was scanned 32 times.

#### XPS analysis tests

XPS was performed using an ESCALAB 250Xi spectrometer (Thermo Fisher Scientific, New York, NY, USA).

#### UV-vis spectroscopy

A small amount of OMC solution (2–3 mL) was placed in a cuvette and irradiated for 24 h, 48 h or 72 h using a CEL-S500/350 xenon lamp (Beijing Zhongjiao Jinyuan Technology Co., Ltd., Beijing, China), with an AM 1.5 filter and a light intensity of 1000 W m^−2^. The solution was diluted 20-fold and UV transmittance was measured over the range 200–400 nm using a TU-1901 double-beam UV-vis spectrophotometer (Beijing Universal Instruments Co., Ltd., Beijing, China), with a scanning step of 1 nm, and ultrapure water as the reference.

#### Storage stability

The OMC/OPCs composite microcapsules emulsion was placed in a glass Petri dish and the surface was covered with plastic wrap to prevent contamination. The sample was stored at room temperature, and the appearance of the sample (front view and side view) was recorded every 30 days using the camera on a Huawei Nova 3 smartphone to monitor the occurrence of demulsification and/or precipitation.

#### Safety

The microencapsulated emulsion was evenly applied to the skin on the inner side of the subject's left wrist and the skin condition was observed after 24 h to check for irritation and swelling.

## The ethic statement on the human test

The microcapsule emulsion prepared in this experiment complies with the “Hygiene Specifications for Cosmetics” and the “Declaration of Helsinki”. Metal elements are not contained in the microcapsules. All subjects participate voluntarily and the experiment fully respects the personal wishes of the subjects. Meanwhile, the experiment is carried out under the supervision of the supervisor and the school safety committee, it is ready to accept ethical review at any time.

## Conflicts of interest

The authors declare no conflict of interest.

## Supplementary Material

RA-011-D0RA09116B-s001
