# Supplementary material for: Simple, green, ultrasound-assisted preparation of novel core–shell microcapsules from octyl methoxycinnamate and oligomeric proanthocyanidins for UV-stable sunscreen
Source: RSC Adv. 2021 Feb 4;11(11):6374–82. doi: 10.1039/d0ra09116b (PMC8694810; doi:10.1039/d0ra09116b)
Supplement: RA-011-D0RA09116B-s001 [file RA-011-D0RA09116B-s001.pdf]

Electronic Supplementary Material (ESI) for RSC Advances.
This journal is © The Royal Society of Chemistry 2021

Simple, green, ultrasound-assisted preparation of novel core-shell microcapsules from octyl methoxycinnamate and oligomeric proanthocyanidins for UV-stable sunscreen

Jie Songa, Siqi Chena, Xu Zhaoa, Junbo Chenga ,Yanli Maa, Shixue Rena,*, Shujun Lia

a. Key Laboratory of Bio-Based Material Science & Technology (Northeast Forestry University), Ministry of Education, Harbin 150040, China

Supplementary Data

The OPCs and proanthocyanidinB1 were characterized by hydrogen nuclear magnetic resonance spectroscopy (NMR), as shown in Fig. S1. (The standard proanthocyanidin is a typical dimer composed of catechin and epicatechin.) The 1H NMR spectra of OPCs and proanthocyanidin B1 show peaks at 1.9 ppm, 2.5–3.3 ppm, 3.6–4.4 ppm, 4.6–5.0 ppm, 5.7–6.0 ppm, 6.1 ppm , 7.7-8 ppm and 8.8–9.2 ppm. 33,39 The spectrum of OPC is similar to those of proanthocyanidin B1, showing that the structure of the extracted product are similar to those of proanthocyanidin B1.

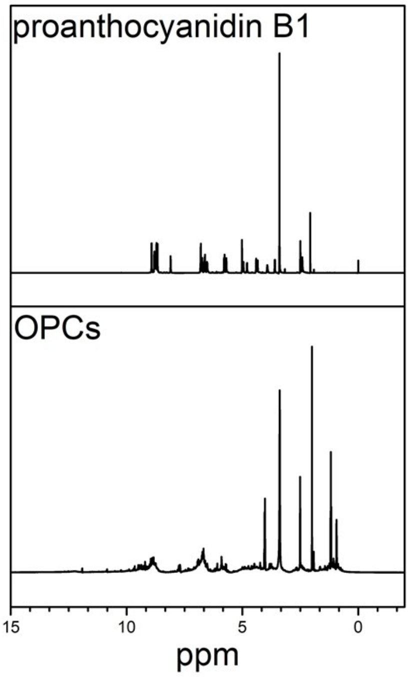

Fig. S1. ^{1}H NMR spectra of oligoproanthocyanidins and standard proanthocyanidins B1

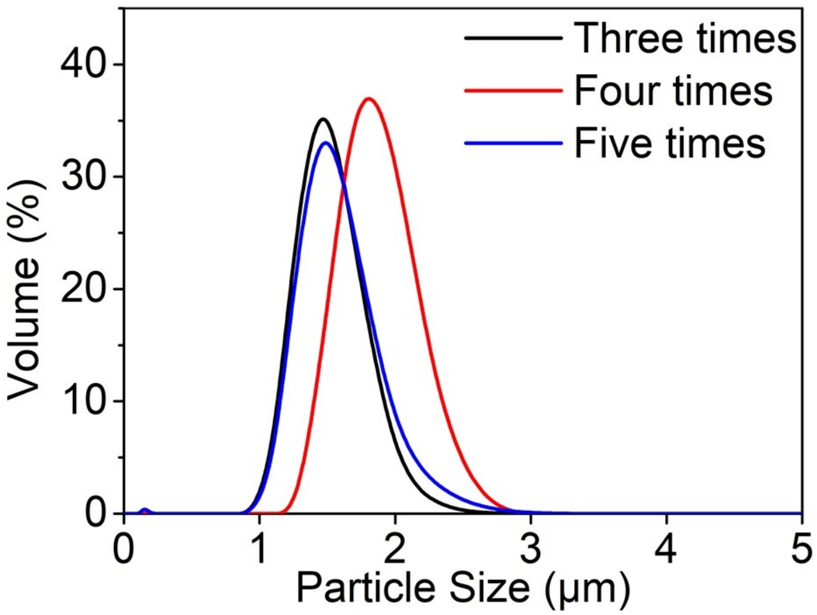

Fig. S2. Particle size distribution diagram of the microcapsules after repeated centrifugation and redispersion

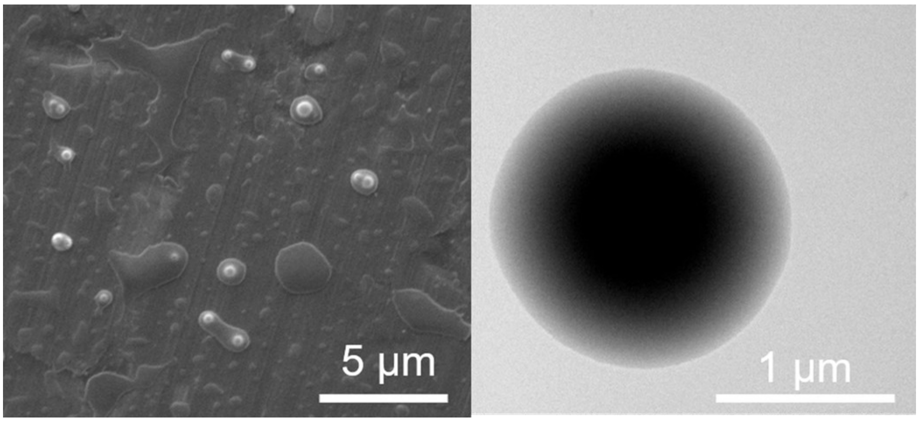

Fig. S3. SEM and TEM image of microcapsules without surfactant added.
Supplementary Information

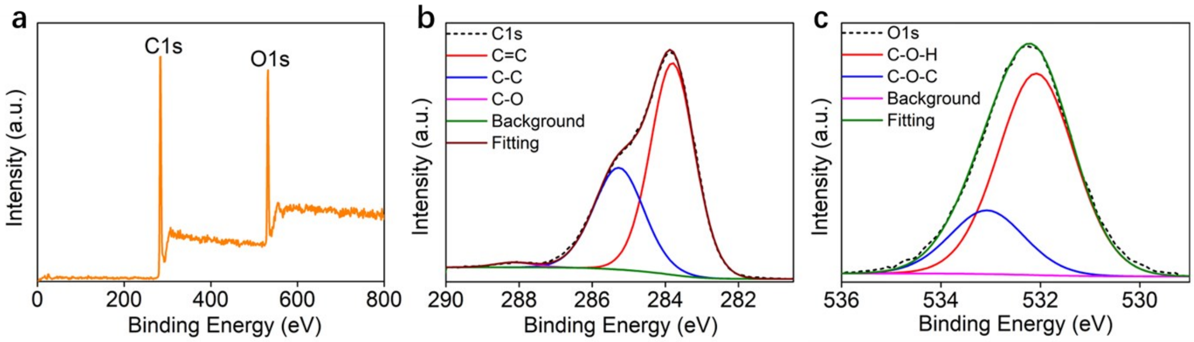

Fig. S4 (a) Full XPS spectrum of OPCs; (b-c) O1s and C1s XPS spectra of OPCs
